# The Possibilities and Limits of “Co-producing” Research

**DOI:** 10.3389/fsoc.2019.00023

**Published:** 2019-04-05

**Authors:** Jonathan Paylor, Christopher McKevitt

**Affiliations:** School of Population Health and Environmental Sciences, King's College London, London, United Kingdom

**Keywords:** co-production, public participation, knowledge production, articulation, impact, knowledge economy, performativity, consumerism

## Abstract

In this perspective paper, we explore the growing enthusiasm for “co-produced” research, focusing in particular on the United Kingdom's National Institute for Health Research's (NIHR) recent adoption of the term co-production. We consider how this interest in co-production is driven by concerns that patient and public involvement (PPI) in health research tends to be “tokenistic” and to reproduce power imbalances between researchers and lay contributors. We argue that these apparent implementation “barriers” or “inconsistencies” need to be understood in relation to the various elements that the institutionalisation of PPI brings together. We show how these elements are articulated in such a way that consumer, managerial, and performative logics and practices are dominant, resulting in limits being placed on the scope and forms of PPI, and the emergence of acts of recalcitrance and impression management. By considering the alternative discursive repertoires made available through co-production, we point to the possibilities co-production presents for moving beyond these dominant tendencies. We argue, however, that such possibilities need to be understood in relation to the constraints of the present. In doing so, we draw attention to the tenacity of the articulations that have historically constituted the institutionalisation of PPI.

## Introduction

Indicative of a heightened interest in promoting participatory approaches to knowledge production, the term “co-production” has recently entered the lexicon of research funders in the United Kingdom (Bell and Pahl, 2017). The UK's National Institute for Health Research (NIHR) is one funder that appears to be particularly enthused by co-production. In *Going the Extra Mile* (NIHR, 2015a), co-production is placed at the heart of the NIHR's plans to “improve” public participation in health research [what the NIHR have traditionally referred to as “patient and public involvement” (PPI)]. As the emphasis on “sharing of power” (Hickey et al., 2018, p. 7) indicates, the NIHR frames co-production as offering a more collaborative and egalitarian mode of involvement than that of conventional PPI approaches. In this perspective paper we ask whether the embracement of co-production will translate into the enhanced forms of involvement the NIHR speaks of. To do so, we draw on insights from an analysis of relevant policy and practice guidance documents as well-previous ethnographic work (Fudge et al., 2008; McKevitt et al., 2010; Komporozos-Athanasiou et al., 2016).

Our starting point is to examine the potential reasons for the NIHR's enthusiastic adoption of co-production. We suggest that in part this stems from concerns about PPI being “tokenistic” and power remaining firmly in the hands of researchers. In seeking to counter previous literature which suggests that these implementation “barriers” or “inconsistencies” are simply down to the negative attitudes or inabilities of researchers, we bring to the fore the contextual conditions of their existence. We do so by exploring how the institutionalisation of PPI is articulated (Slack, 1996) in such a way that consumer, managerial and performative logics and practices are dominant. We show how this entails limits being placed on the scope and form of PPI and the emergence of acts of recalcitrance and impression management. By considering the alternative discursive repertoires made available through co-production, we point to the possibilities co-production presents for moving beyond these dominant tendencies. We argue, however, that such possibilities need to be understood in relation to the constraints of the present. Here we draw attention to the tenacity of the articulations that have constituted the institutionalisation of PPI, arguing that the alternative discursive repertoires offered through co-production are likely to be articulated with and subordinate to the consumer, managerial and performative ways of thinking and acting that have historically imbued PPI.

## The Shortcomings of PPI?

The NIHR's interest in co-production appears to be driven by concerns that existing PPI practice falls short of generating the collaborative forms of involvement that the NIHR and other proponents of PPI seek. While it is reported that there is “much to celebrate” (NIHR, 2015a, p. 15) concerns are expressed about “[i]nconsistencies in practice and implementation and [b]arriers to public contributing to research including negative attitudes and lack of support” (NIHR, 2015a, p. 21). The references to scepticism and paternalism (NIHR, 2015a, p. 37) implies that much of this has to do with the recalcitrance or lack of commitment on the part of researchers. Recommendations to improve infrastructure and support reveal a strategy for overcoming these issues that is broader than co-production alone. Nevertheless, co-production—with its proclaimed ability to “encourage collaboration and underline the value of people's expertise through experience” (NIHR, 2015a, p. 12)—is framed as the potent force that could bring about the necessary change in attitudes and practices and ultimately help to deliver research that improves the “health and wealth of the nation” (NIHR, 2015a, p. 12).

The “authoritative instrumentalism” (Shore and Wright, 2011, p. 4) that underpins the NIHR's approach to PPI is clear to see in *Going the Extra Mile*. Both PPI and co-production are presented as means to solve a problem and produce a desired outcome. They are also portrayed as “things” to be implemented and managed. This “orthodox” approach to policy also frames much of the literature on PPI, not least in the numerous studies on barriers and enablers, impact and good practice (see, for example, Brett et al., 2014). These texts often place tokenism and other implementation “failures” at the hands of researchers who are reluctant to share power and/or recognise the value of “lay” knowledge. One problem with such accounts is that they tend to instil a narrow view of implementation that filters out the diverse ways in which actors interpret and reconstitute policy and ignores how such processes of “translation” (Clarke et al., 2015) involve struggles over subjectivity and power. Moreover, the contextual conditions of these apparent implementation inconsistencies and failures are seldom reckoned with. As we now move to show, the tensions and antagonisms that surface in PPI practices are not simply the product of negative attitudes nor discordant actors but rather the combination of multiple social forces.

## Articulating PPI

We want to draw attention to three trends and trajectories that are important to understanding how the policy of PPI has been institutionalised and enacted. The first is the rise of consumerism. The figure of the consumer came to prominence following Thatcher's neoliberal public-sector reforms. Key to the mobilisation of these reforms was the notion that the public had to be saved from an overbearing and unresponsive state—a motif which saw the government create “synapses” (Clarke et al., 2007, p. 29) between the disaffections and demands of social movements and user groups, and the neoliberal logics of choice and market competition (Cowden and Singh, 2007). New Labour brought a renewed emphasis on public service values and deliberative democracy, heralding a move to animate conceptions of the citizen. It is within this context that a system of “Patient and Public Involvement” was introduced in the NHS and the “Standing Advisory Group on Consumer Involvement in the NHS Research & Development Programme” was renamed INVOLVE. While such developments have been described as an attempt to “put right the failings of the overtly consumerist approach to involvement” (Martin, 2007, p. 42), the image of the consumer remained a fundamental feature of New Labour's discourse and provided “the central element around which the other, subordinate, elements were articulated” (Clarke et al., 2007. p. 44). More recent reforms relating to PPI came with the Coalition government's Health and Social Care Act (Department of Health, 2012). Patient choice and market competition were at the centre of these reforms, signalling the continual prominence of the consumer figure.

The second is the advancement of “performativity.” Predicated on the drive for efficiency and greater productivity, the neoliberal public sector reforms also gave rise to management practices based on individualised incentives and the measurement of performance. Exemplified by the onset of quasi-market mechanisms and quality audits such as the Research Assessment Exercise (RAE) (Elton, 2000), this move toward systems of performativity had a notable bearing on the organisation and governance of universities. Along with the “publish or perish” imperative, one of the consequences of this shift is the increased pressure on academics to secure external research funds. As some have suggested (Chubb and Watermeyer, 2016), a “grants culture” now dominates UK universities as academics strive to remain economically viable and prove their worth through the procurement of funds. The establishment of the NIHR played a notable role in fostering such a grants culture in the health domain. Prior to the NIHR's conception, funding was locked into historical allocations to NHS trusts (Shergold and Grant, 2008). Premised on the goals of funding the “best research” and “acting as sound custodians of public money for public good” (Department of Health, 2006), the NIHR centralised this funding and made it available through various competitive funding streams. Crucially, this reorganisation of research funding put PPI further into the limelight as it became a condition of funding and an assessment criterion.

The third is the shift to a global knowledge economy. This transition has seen the UK health research system and the NHS become a central feature in the government's plans to build the nation's knowledge economy, as exemplified by the creation of the NIHR and its vision to improve the “health and wealth of the nation” (Department of Health, 2006). This health and wealth agenda brings together a wide range of elements (including different logics, values, technologies and actors) which are articulated around the aim of speeding up knowledge translation (Adams and McKevitt, 2015; Caffrey et al., 2018). Alongside the push for “stakeholder” involvement (including patients and industry) this move to align health research with the imperatives of a global knowledge economy has resulted in a greater emphasis on biotechnology and microbiological sciences as well as a drive for clinical trials (Shaw and Greenhalgh, 2008).

## Tendencies in PPI Practices

A consideration of these three trends and trajectories starts to uncover the multiple and diverse elements that the policy of PPI and its institutionalisation articulates. The coming together of these different elements complicates readings that simply frame PPI as the product and manifestation of neoliberalism or any other dominant force. Such multiplicity, however, does not mean dominant tendencies do not exist. The various elements that are brought together are “structured in dominance” (Newman and Clarke, 2009, p. 26) and articulated in ways that shape the possibilities for thinking and acting. What we are particularly interested in here is how this offers an insight in to the formation of power differentials and the surfacing of “tokenistic” PPI.

One tendency is the dominance of consumerist or managerialist models of PPI. While the spaces that institutionalisation of PPI generates are diverse and contain possibilities for multiple forms of action, PPI practices tend to reproduce processes of consultation that position the public as individual consumers rather than democratic publics. In such settings, the rationalities of funders and research teams delineate the scope of involvement. This often means that PPI is merely a tool to gather feedback on the relevance and appropriateness of predefined research aims and procedures. Thus, decision-making tends to remain in the hands of researchers, and issues deemed out of scope are side-lined. Moreover, viewed in light of the linkages with systems of performativity and the goal of speeding up knowledge translation, such contained involvement can be seen to embody and instil a mode of public accountability that is narrowly defined in terms of efficiency and cost-effectiveness.

The dominance of consumerist and managerialist models of PPI goes some way to understanding why concerns are expressed about the restricted nature of PPI and the reproduction of power imbalances. It's also important to consider the type of research that the NIHR funds. As exemplified by the “gold standard” of the randomised controlled trial, NIHR-funded research tends to adhere to the tenets of positivism and thereby embed a knowledge hierarchy that privileges scientific expertise over lay understandings. It's no surprise then that the remit of PPI often centres on the appropriateness of trial procedures and materials such as patient information sheets. As valuable as this may be, especially if trial recruitment is a priority (Adams and McKevitt, 2015), it does point to a situation where the various components and phases of a study are controlled and undertaken by researchers. It's also important to consider how the NIHR funds research. Much of the NIHR's funding is allocated on a study-by-study basis and thus feeds into processes of “projectisation” (Newman and Clarke, 2009, p. 150). In this way, PPI often becomes a bounded event which operates in line with the managerial logics of the health research system. Coupled with the temporal pressures of the “accelerated academy” (Carrigan, 2015), we suggest that such time-limited involvement hinders collaboration and sustained dialogue.

The allocation of grants brings us onto how PPI becomes enmeshed in a “grants culture.” We want to suggest that making PPI a condition of funding and an assessment criterion has three consequences^1^. Firstly, PPI adds to the multitude of activities that researchers need to perform to maintain and advance their career, generating tensions and acts of recalcitrance or resistance as researchers find themselves having to negotiate different demands and logics. Secondly, PPI is performed to meet the requirements and expectations of funders. This may manifest itself in surface level spectacles or acts of impression management, which are deemed an inevitable part of “playing the game” and securing research funds. Thirdly, PPI destabilises researchers' professional identity, as their enactment of PPI is imposed rather than based on their own judgment. This destabilisation may also be brought on by the undermining of integrity and collegiality that results from needing to succeed in the competitive game of grant-seeking and perform acts of dramaturgy.

These three points bring into further focus the reasons why PPI may be described as “tokenistic.” They also cast concerns about sceptical or recalcitrant researchers in a new light. Rather than simply reflecting ingrained paternalist or elitist views, researchers' identifications with traditional conceptions of professionalism may signify an attempt to deal with the destabilisations and costs that are induced through the regimes of performativity that PPI embodies and augments. Such processes of identity formation perhaps reveal a “struggle over subjectivity” and a “politics of refusal” (Ball, 2016); that is to say, the refusal to take up the subject positions made available through discourses of performativity. To be clear, we do not wish to downplay the existence and regressiveness of paternalism or elitism, nor to suggest that the redrawing of traditional professional-lay boundaries amounts to effective forms of resistance and mobilisation. Rather, our contention is that the reasons that sit behind “negative attitudes” are far more multifaceted than the NIHR and much of the literature on PPI suggests.

## Articulating PPI With co-production

What possibilities does “co-production” present for moving beyond the tendencies outlined above? The first thing to note is that while the NIHR frames co-production as distinct from existing understandings and practices of PPI, both PPI and co-production are concepts that have historically been interpreted in various—and indeed analogous—ways. It's understandable if some think co-production is “old wine in new bottles” (Hickey et al., 2018). Nevertheless, we want to suggest that the uptake of term co-production could expand the discursive repertoires available to actors, generating possibilities for forms of involvement that transcend dominant understandings and practices.

What is perhaps most significant is how co-production could encourage (re)engagement with participatory research traditions and “bottom up” social movements that bring questions of empowerment, ethics and social justice to the fore. This may help to dislodge the consumerist and managerialist tendencies of PPI, aligning it more with democratic currents that centre on deliberative and collective forms of involvement. Such a realignment could “offer connections between publics rather than further processes of individuation” (Newman and Clarke, 2009, p. 151). It could also open up a space where broader questions of public legitimacy and social transformation are addressed, providing the grounds for a mode of accountability that isn't simply narrowly defined in terms of using public resources efficiently and effectively.

Such possibilities, however, exist within the constraints of the present. What we are particularly concerned with here is how the articulations that provide the conditions for the tendencies in PPI practice discussed earlier exhibit what Stuart Hall (Grossberg, 1996) called “lines of tendential force.” That is to say, “they are rather firmly forged and difficult to disarticulate” (Slack and Wise, 2005, p. 128). The tenacity of consumerism can be seen in recent attempts to introduce co-production models of public service design and delivery. For instance, Glynos and Speed (2012) show that a mode of co-production based on the logics of cooperation, generalised reciprocity and collective deliberation sits uncomfortably with and is subordinate to a health care regime centred around choice. The tenacious force of the knowledge economy is evident in the continuation of the NIHR's health and wealth agenda (NIHR, 2015b). And, as *Going the Extra Mile* indicates (2015a, p. 12), it is these rationalities that underpin their interest in co-production.

A culture of performativity also shows no sign of abating. A significant development is the recent policy drive to assess the impact of research beyond academia. This is exemplified by the Research Excellence Framework (REF) succeeding the RAE—a move which signifies a more pronounced connection between the imperatives of a global knowledge economy and systems of performativity (Holmwood, 2014). Importantly, it is within this context that co-production has gained currency and has been framed as a way to help “shorten the ‘time from idea to income’ or the research-development cycle” (Holmwood and Balon, 2018, p. 309). Another notable development relating to performativity is the NIHR's desire to develop a set of standards to assess and improve PPI (NIHR, 2015a, p. 17). This coincides with the recommendation to measure the success of PPI and to develop its “evidence base for REF2020” (NIHR, 2015a, p. 18).

These tendential lines of force shift attention to the likelihood of alternative discursive repertoires offered by co-production being articulated with and subordinate to the consumer, managerial and performative logics that have historically imbued PPI. We thus want to offer a note of caution and suggest that it cannot be assumed that the change in signifier (“co-production”) will lead to greater collaboration and “sharing of power.” As we have shown, these dominant logics tend to give rise to narrowly defined and “thin” forms of involvement that curb how the public can be involved and what they can say. Moreover, there is a risk that the shift towards co-production could exacerbate the tensions that surface in PPI practices. The public may become more dissatisfied as they are promised greater power that fails to materialise in practice, while researchers may feel pressurised to perform “co-production” to meet expectations and “standards,” furthering acts of dramaturgy and resistance.

We want to close by underscoring the need to remain open to the ambivalent potentialities of the turn to co-production. As previous ethnographies have shown (Komporozos-Athanasiou et al., 2016), the institutionalisation of PPI generates ambiguous spaces where enactments of PPI exceed binary distinctions (such as empowerment vs. consumerism) and where the form and outcomes of such enactments cannot be predicted in advance. It's likely that the arrival of co-production will augment such ambiguities, and researchers need to be attentive to how they unfold in everyday practices.

## Author Contributions

JP drafted the manuscript. CM provided feedback on drafts.

### Conflict of Interest Statement

The authors declare that the research was conducted in the absence of any commercial or financial relationships that could be construed as a potential conflict of interest.

## Footnotes

^1^The argument we make here draws particularly on a study conducted by ourselves and colleagues which used in-depth interviews to explore researchers' views and experiences of PPI.
